# Complete mitochondrial genome of *Laqueus Japonicus* (Brachiopoda, Terebratulida, Laqueidae)

**DOI:** 10.1080/23802359.2017.1407716

**Published:** 2017-11-27

**Authors:** Mustafa Zafer Karagozlu, Seong-Geun Kim, Thinh Do Dhin, Chang-Bae Kim

**Affiliations:** Department of Biotechnology, Sangmyung University, Seoul, Korea

**Keywords:** Brachiopoda, Terebratulida, Laqueidae, complete mitogenome, *Laqueus japonicus*

## Abstract

In this study a complete mitochondrial genome of the species, *Laqueus japonicus* was sequenced and analysed. The mitochondrial genome size is 14,267 bp with 20.2% A, 15.7% C, 27.1% G, and 37.0% T nucleotide distributions. This is the second complete mitochondrial genome record from the genus *Laqueus* and first record for the species. Genome structure and gene orientation are identical with previous record of the genus. In addition, phylogenetic relationship of *L. japonicus* in the subphylum Rhynchonelliformea was investigated by using protein coding genes of complete mitochondrial genome. The present study suggests that the closest species to *L. japonicus* is *L. rubellus* and they belong to the family Laqueidae.

The phylum Brachiopoda are relict marine invertebrates with approximately 4500 named genera grouped into 109 superfamilies, 26 orders, eight classes, and three subphyla (Carlson 2001; Drozdov et al. 2015). Among these three, the Rhynchonelliformea is the most diverse subphylum (WoRMS Editorial Board 2017). Despite species richness there are only three complete mitochondrial genomes recorded from the subphylum Rhynchonelliformea which are *Terebratulina retusa* (Stechmann and Schlegel 1999), *Terebratalia transversa* (Helfenbein et al. 2001) and *Laqueus rubellus* (Noguchi et al. 2000). In this study, complete mitochondrial genome of a rhynchonelliformean species *Laqueus japonicus* (Yabe and Hatai 1936) analysed and compared with previous record from the same genus. This is the first record for the species.

The *L. japonicus* specimen was collected in January 2002 from Samcheok, Gangwon-do, South Korea (37°13'44" N, 129°21′36″ E) and deposited in the Department of Biotechnology, Sangmyung University, Korea University (SM00014) after identified by COI barcoding. The mitochondrial DNA was extracted from whole body of the specimens. For mitochondrial genome sequencing and phylogenetic tree analysis, the methods that already described in the previous study were followed (Karagozlu et al. 2016).

Mitochondrial genome of *L. japonicus* is 14,267 bp in length (GenBank accession number: MG520333). Although size of the mitochondrial genome is slightly longer than *L. rubellus* (14,017 bp), their genome structure and gene orientation are identical. The main reason of the difference is a long non-coding area between tRNA-His and ATP6 gene in the *L. japonicus* mitogenome. Besides, *L. japonicus* mitogenome consists of 13 protein-coding genes, two ribosomal RNA and 22 tRNA and all genes encoded on the majority strand these structural features are identical as well. On the other hand the nucleotide composition of the genome is 20.2% A, 15.7% C, 27.1% G, and 37.0% T. There are 18 overlapping regions in the genome and 11 intergenic sequences show length variation ranging from 2 to 286 bp. In the mitogenome the genes were formed using five different starting codons: ATA, ATG, ATT, TTG and CAA. The most common starting codon is ATT that was used by ATP8, NAD3, NAD4L, NAD5, and NAD6 genes. Also, there were four different stop codons observed: TAA, GTT, TAG, and incomplete T(AA). The most common stop codon is TAG that was used by ATP8, COX1, NAD1, NAD3, NAD4, NAD5, and NAD6 genes.

The phylogenetic relationships of *L. japonicus* in the subphylum Rhynchonelliformea were investigated (Figure 1). The reconstruction of the phylogenetic tree suggests that *L. rubellus* is the closest species to the *L. japonicus* and they are belonging to the monophyletic family Laqueoidea. The previous mitochondrial gene (COI) study and nuclear small subunit rRNA gene-based studies also declared the similar results (Carlson 2016; Cohen et al. 1998; Saito et al. 2000). The lack of information on complete mitochondrial genome of the species infers to confirm exact location of the *L. japonicus* in phylogenetic tree. For further studies complete mitochondrial genome data records should increase to investigate molecular phylogeny. This study provides additional data for the subphylum Rhynchonelliformea phylogeny.

**Figure 1.**
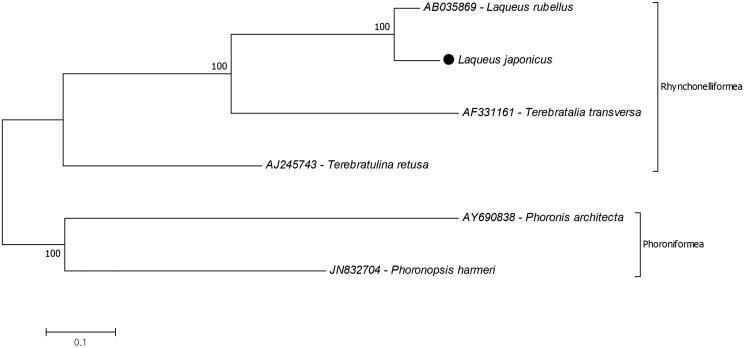
Molecular phylogeny of the *Laqueus japonicus* in the subphylum Rhynchonelliformea. The two species from the subphylum Phoroniformea represent outgroup. The phylogeny tree reconstructed due to protein coding genes of mitochondrial genome (ATP8 excluded) with maximum likelihood statistical method by using MEGA 7.0 software. mtREV with Freqs (+F) model used for amino acid substitution and bootstrap method replicated 1000 times for the test of phylogeny. The complete mitochondrial genomes were retrieved from the GenBank. *Laqueus japonicus* data marked with a black dot.
